# 

**Published:** 1959-10

**Authors:** 


### THE MEDICAL JOURNAL OF THE SOUTH-WEST

*Incorporating*

*The Bristol Medico-Chirurgical Journal*

NATIONAL LIBRARY OF MEDICINE
FEB 3 1960

*October 1959*

*January 1960*

